# Motor Coordination Disorders in Patients with Chronic Kidney Disease

**DOI:** 10.3390/jcm14082804

**Published:** 2025-04-18

**Authors:** Patryk Jerzak, Mariusz Kusztal, Wioletta Dziubek, Łukasz Rogowski, Bożena Ostrowska, Maciej Gołębiowski, Paulina Bronikowska, Maria Chumadevska, Jakub Stojanowski, Tomasz Gołębiowski

**Affiliations:** 1 Clinical Department of Nephrology, Transplantation Medicine and Internal Diseases, Wroclaw Medical University, 50-367 Wroclaw, Poland; patryk.jerzak@umw.edu.pl (P.J.); chumadevska28@gmail.com (M.C.); jakub77xx@gmail.com (J.S.); tomasz.golebiowski@umw.edu.pl (T.G.); 2 Faculty of Physiotherapy, Wroclaw University of Health and Sport Sciences, 51-612 Wroclaw, Poland; bozena.ostrowska@awf.wroc.pl; 3 Faculty of Health and Physical Culture Sciences, Witelon Collegium State University, 59-220 Legnica, Poland; lukasz.rogowski@collegiumwitelona.pl; 4 Faculty of Medicine, Wroclaw Medical University, 50-367 Wroclaw, Poland; maciej.golebiowski@student.umw.edu.pl (M.G.); paulina.pabisz98@gmail.com (P.B.)

**Keywords:** coordination disorder, falls, CKD, vessel stiffness

## Abstract

**Background**: The number of senior chronic kidney disease (CKD) patients is steadily increasing worldwide. Falls are more frequent in this group than in the general population, and they are associated with a variety of complications ranging from minor (bruises) to severe (fracture, brain injury, or death). The significant burden of comorbidities, particularly cardiovascular disorders, impacts coordination. The aim of the study was to assess coordination disorders in CKD patients in the context of cardiovascular complications and vascular status. **Methods**: In this prospective study, 132 patients with CKD 2–5, including 40 (30%) hemodialysis patients, were enrolled. The short form physiological profile assessment (S-PPA) was used to assess coordination. **Results**: During a 2-year follow-up period, 49 individuals experienced 84 falls. The median S-PPA score (Z score) was 3.36. Based on this, we divided our cohort into two groups: a Z score of <3.36 and a Z score of ≥3.36. The groups with high scores (≥3.36) characterized by higher parameters of vessel stiffness, including AIx@75, augmentation pressure, and PWV, experienced considerably greater numbers of falls (41 vs. 8, *p* < 0.001), CV events (10 vs. 2, *p* < 0.05), and deaths (14 vs. 0, *p* < 0.001). **Conclusions**: Coordination impairments and the associated risk of falls in CKD patients are directly related to cardiovascular diseases and vascular conditions. Lower arterial compliance has been linked with the largest coordination disorder. Visual impairments, especially contrast sensitivity, are an independent risk factor for falls.

## 1. Introduction

Falls are a significant concern for chronic kidney disease patients (CKD). Studies have shown that individuals with CKD have a higher risk of falling compared to those without the condition. This increased risk is due to several factors, including muscle weakness, balance issues, and complications related to bone health [1,2]. This increased risk is the result of the co-occurrence of many diseases and systemic disorders with CKD, generating several complications, including muscle weakness and balance issues. Falls in CKD lead to serious injuries, such as fractures, which, due to impaired bone metabolism, are 2–4 times more common compared to people without CKD. The most dangerous fracture, hip fracture, is associated with immobilization, loss of independence, disability, and high mortality [2]. Repeated falls contribute to the development of post-fall syndrome, causing reduced physical activity, social isolation, and thus an increased risk of falling [2].

The prevalence of fall accidents was 24.7% among patients with CKD, compared to 17.1% in those without CKD. Additionally, falls in CKD patients can lead to serious injuries, such as fractures, which further impact their quality of life [2]. Falls in patients can lead to significant costs, both direct and indirect. Falls and the injuries they cause, apart from the health consequences, generate significant social costs. Direct costs include medical expenses such as hospitalizations, treatments for injuries (like fractures), rehabilitation services, and admission to nursing homes. Indirect costs can involve lost productivity, long-term disability, and the emotional and psychological impact on patients and their families. For instance, in the United States, the cost of falls was estimated to be around USD 30 billion in 2010. While this figure includes the general population, CKD patients are at a higher risk of falls, which can lead to more frequent and severe injuries, thereby increasing the overall costs [2].

Regardless of the presence of disease risk factors for falls, their incidence in the general population increases with age. About one-third of people over 65 years of age fall at least once a year, with CKD significantly increasing this risk, which ranges from 1.18 to 1.60 falls/patient per year [3,4,5]. The risk of falls is particularly high in elderly patients and in advanced stage 5 kidney disease, culminating in dialysis patients, where orthostatic hypotension may also occur after dialysis. The etiology of falls is multifactorial, ranging from decreases in cerebral blood flow, increased arterial stiffness, decreased muscle mass and strength, joint dysfunction, cognitive impairment, weakness and fragility syndrome, to depression and insomnia. Among the mentioned impairments, the contribution of vascular disorders to the course of CKD as a predisposing factor to postural instability and increased susceptibility to falls has not been fully investigated [3,4,5].

Fall prevention in CKD requires coordinated actions of a multidisciplinary team, including reliable measurement of risk factors related to health and functional status. In previous studies, tests that include balance, muscle strength, and mobility, such as the timed up-and-go test, Berg balance scale (BBS), functional reach test (FRT), or short physical performance battery (SPPB), were most often used to assess fall risk. However, these measures have certain limitations in terms of their psychometric values and the ability to capture fall risk factors such as peripheral sensation, vision, or reaction time to stimuli.

Lord et al. [6] proposed a physiological measure of fall risk to examine sensorimotor performance and balance in older people (short form physiological profile assessment; S-PPA) [6]. The main advantage of S-PPA is that falls are understood as an interaction of a number of sensorimotor factors, not just the efficiency of the individual’s balance system, demographic factors, or clinical characteristics. PPA has been used so far in studies of the elderly population, including people with various chronic diseases, such as stroke, Parkinson’s disease, multiple sclerosis, or chronic obstructive pulmonary disease. This tool has been found to be a reliable and valid, yet simple, test for determining physiological dysfunctions contributing to falls, as well as for monitoring the risk of falls and the effects of interventions aimed at impaired functions.

The aim of our research was to explore the potential association between vascular disorders and sensorimotor parameters crucial for motor coordination, which could contribute to an increased risk of falls and mortality in CKD patients.

## 2. Materials and Methods

### 2.1. Participants

The research was conducted at the Department of Nephrology and Transplantation Medicine at the University Clinical Hospital in Wroclaw. In this prospective study, 132 patients (mean age 64.6 ± 14.6 years; 51 females) with CKD 2–5, including 44 (33%) hemodialysis patients, were enrolled. Study participants were selected from patients diagnosed with chronic kidney disease in stages 2–5 (including dialysis patients) hospitalized in the clinic between March and September 2021.

The study was conducted in accordance with the Declaration of Helsinki and approved by the Ethics Committee at Wroclaw Medical University (No. KB 587/2018).

All patients were informed about the aim and methods of the study and the procedures used. All participants signed a document of voluntary and informed consent.

The inclusion criteria for hemodialysis patients were as follows: (1) age 18 years and over, (2) have stable CKD in stage 2–5 for 3 months, (3) possibility of moving without the need to use additional devices such as a cane, wheelchair, etc., and (4) were able to provide informed consent. The exclusion criteria included the following: (1) currently enrolled in another study, (2) receiving emergency in-patient care within four weeks, (3) unable to walk without additional devices. Written informed consent was received from each patient entering the study. Of the 142 eligible participants, 10 did not agree to take part in the study. Patients who were not recruited were similar in age and gender to those participating in the study. The demographic data and the causes of CKD were collected from medical records and from a direct interview.

### 2.2. Outcome Measurements

#### 2.2.1. The Charlson Comorbidity Index (CCI)

CCI was used to quantify comorbidities and is a validated method used to classify comorbid conditions that might alter the risk of mortality or outcomes in patients. The index assigns a weighted score to a range of comorbid conditions, with higher scores indicating a greater burden of disease and an increased risk of mortality. The CCI involves the systematic review of a patient’s medical history and assigns weights to specific comorbid conditions based on their severity and potential impact on patient outcomes. Each condition is assigned a score between 1 and 6 based on the relative risk of mortality associated with that condition. The final index score is the sum of all individual scores for a given patient [7].

This study assessed the incidence of cardiovascular disease (CV), which included diseases of the heart, vascular diseases of the brain, and diseases of blood vessels, particularly: myocardial infarction; heart failure New York Heart Association (NYHA) class > II, atrial fibrillation; implantable medical devices (IMD), i.e., cardio-stimulator or cardioverter–defibrillator; ischemic or hemorrhagic stroke; amputation due to extremity ischemia or diabetic foot; and surgery to repair an aortic aneurysm.

#### 2.2.2. The Short Form Physiological Profile Assessment (S-PPA)

S-PPA is a streamlined version of the full physiological profile assessment (PPA), which is a validated tool designed to assess an individual’s physical capabilities related to balance, strength, reaction time, and vision. The S-PPA is utilized primarily to evaluate fall risk with specific emphasis on components of physical function that are critical for maintaining postural control and stability. Results from each test are combined to generate a composite score. This score is indicative of the individual’s overall physical performance and potential risk of falling. Higher scores represent better physical function and lower fall risk, whereas lower scores suggest greater impairment and higher fall risk. A detailed description of the test performed was described by Lord and colleagues [6].

#### 2.2.3. Description of the Five Components of S-PPA

Visual Contrast Sensitivity

Contrast sensitivity was measured using the Melbourne edge test, consisting of 20 circular patches with decreasing contrast and varying edge orientations. The lowest correctly identified contrast is recorded as the participant’s sensitivity in decibel (dB) units. The lower the ability to detect contrast, the lower the final Melbourne edge test value in decibels.

Proprioception

Proprioception was assessed using a lower limb-matching task, where participants, with eyes closed, aligned their legs on either side of an acrylic panel. The alignment difference is measured in degrees, averaged over five trials.

Muscle Force Test

Quadriceps strength was measured with a digital dynamometer. Participants pushed against a strap placed 10 cm above the ankle. The best of three trials was recorded in kilograms.

Reaction Time Test

Reaction time was measured in milliseconds using a timer with a light stimulus. Participants pressed a switch in response, with the best of ten trials recorded.

Balance Test

Postural sway was assessed using a sway meter attached to the participant’s lower back, recording movements while standing still for 30 s on a foam mat. The pen records the oscillation on a millimeter graph paper attached to an adjustable-height table. The test was conducted with the participant standing on a 15 cm high foam rubber mat, eyes open. Anteroposterior and mediolateral oscillations were recorded [6].

QRISK^®^3 score is an online tool evaluating a 10-year risk of cardiovascular accident, and it was validated for different ethnicities and CKD patients. It takes into account the following factors: age, ethnicity, diabetes, hypertension, smoking, angina or heart attack in a 1st degree relative <60 y, atrial fibrillation, blood pressure treatment, blood pressure and chronic kidney disease, migraine, corticosteroid use, systemic lupus erythematosus, atypical antipsychotic medication, severe mental illness, and erectile dysfunction. The results are presented as a risk of a heart attack or stroke within the next 10 years.

In addition, ambulatory measurements of hemodynamic parameters with the Mobil-O-Graph monitor (Industrielle Entwicklung Medizintechnik und Vertriebsgesellschaft GmbH (IEM)IEM, Stolberg, Germany), which records oscillometric arm blood pressure: systolic and diastolic blood pressure (SBP and DBP), pulse pressure (PP), ejection fraction (EF), cardiac output (CO), and pulse waves. It calculates the augmentation index normalized to 75 bpm heart rate (AIx@75) as a measure of wave reflections and pulse wave velocity (PWV) as a measure of arterial stiffness. All tests were performed before the start of the one-day hemodialysis (HD) sessions in HD patients.

### 2.3. Statistical Analysis

Statistical analysis was performed using standard software (Statistica Version 13.3, StatSoft, Tulsa, OK, USA). Continuous variables between groups were expressed as mean and standard deviation (±SD) and compared using the independent *t*-test or Mann–Whitney U test, based on the normality of the variables, tested using the Kolmogorov–Smirnov test. Categorical variables were expressed as absolute (*n*) and percentage (%) and compared using the χ^2^ test. The relationship between two continuous variables was examined using Pearson’s correlation analysis. The unadjusted and adjusted multivariate Cox regression analysis was presented as an odds ratio (OR; 95% confidence intervals (CI)). A *p*-value < 0.05 was considered significant.

## 3. Results

### 3.1. Baseline Clinical Data

**Figure 1:**
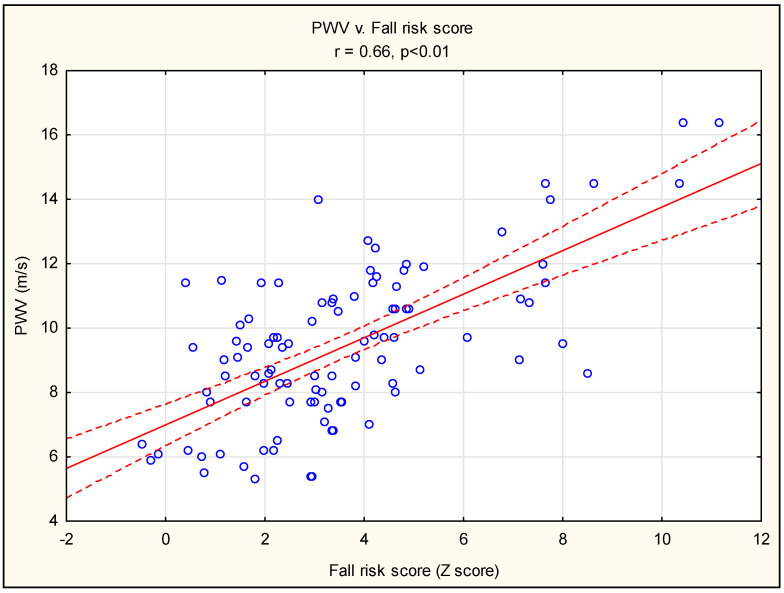
Pearson’s correlation analysis between PWV and fall risk score measured by PPA. Abbreviation: PWV, pulse wave velocity.

The median S-PPA score (Z score) within the study group was 3.36. Based on this, we divided our cohort into two subgroups with Z scores of <3.36 and ≥3.36. Table 1 presents the demographic baselines, PPA test results, hemodynamic parameters, blood chemistry characteristics, and data from a 2-year follow-up. Diabetes and renovascular disease were the leading causes of kidney failure, followed by glomerulonephritis and other conditions; however, there were no differences between the two subgroups. The group with a high score ≥ 3.36 is characterized by significantly higher age, CCI, and higher parameters of vessel stiffness including AIx@75, augmentation pressure, and PWV. Except for proprioception, single tests included in PPA revealed weaker muscle strength in the 30-second sit-to-stand test and knee extension test, elevated postural sway, and a lower ability to contrast discrimination. During the 2-year follow-up period, this group experienced considerably greater numbers of falls (41 vs. 8, *p* < 0.001), cardiovascular (CV) events (10 vs. 2, *p* < 0.05), and deaths (14 vs. 0, *p* < 0.001). During this time, 49 persons fell 84 times, more often than the group of patients with a Z score ≥ 3.36 (41 vs. 8, *p* < 0.01). There were no significant differences regarding laboratory parameters except lower triglycerides in the larger Z score subgroup. In this group, lower weight was also observed, indicating more advanced malnutrition. Patients with a Z score of ≥3.36 had higher hemodynamic parameters of vascular stiffness, both small vessel parameters (AIx@75) and medial vessel indices (PWV or pPP). Pearson’s correlation analysis shows a strong relationship between PWV and PPA score (Z score) (r = 0.66, *p* < 0.01) (Figure 1).

### 3.2. Results of Logistic Regression Model

Table 2 shows the crude odd ratios (ORs) for all predictors. During a 2-year follow-up, variables related to at least one fall included age ≥ 70 years, female sex, greater CCI, pulse pressure (pPP), PWV, higher QRISK^®^3, and all single PPA test values.

No significant relationship with patients who have fallen was observed for BMI, incidence of CV events, AIx@75, and laboratory parameters, including hemoglobin level, total protein, albumin concentration, and phosphate.

### 3.3. Development and Results of Multivariate Logistic Regression Model

Included in the model were variables that were significantly associated with at least one fall during the two-year follow-up time: sex, age ≥ 70, CCI, PWV, pPP, and QRISK^®^3 (Table 3).

Multivariate logistic regression revealed that contrast sensitivity in the Melbourne edge test was independently associated with fall events in the 2-year follow-up and the adjusted odds ratio (adj. OR) was 0.71 (95%CI: 0.53–0.95).

## 4. Discussion

In our two-year observational study, a significant increase in falls in the group of older patients and poorer physical fitness, expressed by lower muscle strength, hand reaction time, and greater postural sway, were observed. As demonstrated in Table 1, this subset of patients had a higher PPA score, which was greater than 3.36. Furthermore, we discovered that cardiovascular events and death were more common among these patients.

Our findings indicate a strong association between arterial stiffness and the risk of falls. The positive Pearson correlation between PVW and PPA score (Figure 1) largely supports this hypothesis. Obtained results are consistent with the studies of other authors in the CKD group [8,9]. In these studies, impaired baroreflex function and orthostatic blood pressure drops, as well as poor muscle strength and physical function, all contribute to the falls. In CKD stage 5 (dialysis) patients, age, comorbidity, dialysis-related hypotension, and cognitive and functional impairment were associated with the risk of fall and mortality [10,11]. CKD is often linked to sensory and motor nerve dysfunction stemming from vascular dysfunction, diabetic neuropathy, and uremic sarcopenia, which collectively can compromise reaction time, balance, and muscular strength [12,13]. Finally, frailty syndrome with sarcopenia and reduced muscle strength is a well-established risk factor for falls [14,15].

In CKD patients, arterial hypertension is commonly observed and accompanied by vascular changes [16,17] Arterial stiffness, assessed via pulse wave velocity (PWV), is linked to an increased risk of cardiovascular disease (CVD) and overall mortality [18,19]. It has been suggested that circulatory disorders may represent a more significant risk factor for falls compared to conditions such as frailty syndrome [6,20]. We observed no differences in blood pressure between subgroups with PPA scores of <3.36 and ≥3.36; however, in the last group (with PPA ≥ 3.36), we noted higher hemodynamic parameters of vascular stiffness, both small vessel parameters (AIx@75) or medial vessel indices (PWV or pPP), and a higher risk of CVD events, as determined by risk calculation (QRISK3). PWV, a marker of arterial stiffness, is known to correlate with age and cardiovascular risk factors. Those factors can influence fall risks, although specifics for CKD populations are scarce. Zanotto et al. noted that a significant proportion of falls appeared to be mediated by a degree of cardiovascular dysregulation with a drop in blood pressure in response to a 60° head-up tilt test. Many fall events were preceded by self-reported dizziness. The authors additionally state that including variables related to cardiovascular function in the frailty model may significantly improve the prediction of falls [6,8,20].

Arterial abnormalities can diminish the efficiency of the central nervous system, contributing to dysregulation of spatial disorientation, delayed reaction time, and poorer balance [21]. In the PPA items that assessed motor coordination, balance, and perception/vision, we noticed poorer scores among older, more burdensome patients with PPA with PPA ≥ 3.36. We have noted a greater postural sway and worse outcomes in the Melbourne edge test (MET) in the group at higher risk of falls. This is consistent with the observations of other research teams and may indicate central causes of balance disorders. One prospective cohort study found that higher postural sway in the medial–lateral direction was associated with increased odds of falling in people receiving HD and have prognostic value in discriminating patients who have fallen from patients who have not fallen [22]. Other studies have identified poor balance, unsteady gait, cognitive deficits, and visual impairments as risk factors for falls in community-dwelling older adults [21,22,23]. Although not all of these factors may have the same impact on patients with CKD, prospective studies suggest that older age, comorbidities, polypharmacy, and autonomic dysregulation contribute significantly to the risk of falls in this population [24,25]. In our study, this is reflected in the observed correlation between pulse wave velocity (PWV) and the fall risk index (PPA), as well as the results of the regression analysis including the fall risk index, mean hand reaction time, and Melbourne edge test (MET) scores.

Notably, the multivariate logistic regression revealed that the MET was an independent predictor of falls. In a study by Wood et al. [26], reduced contrast sensitivity was linked to postural instability, slower walking speed, wider step width, and shorter stride length. It should be noted that individuals with CKD experience a significant burden of visual impairment and severe eye conditions, with nearly 50% affected by low vision [27,28]. Contrast sensitivity declines with age and may even worsen in high-functioning elderly people, potentially increasing the risk of falls, especially when combined with other risk factors [24,28]. According to data in the literature, those with end-stage kidney disease and early stages of CKD are more likely to have eye problems and vision impairment than those without. Among the major eye illnesses, diabetic retinopathy and age-related macular degeneration are most frequently associated with CKD. These disorders may be related to the underlying disease and risk factors for CKD, such as diabetes or hypertension, metabolic disorders associated with CKD, uremia and anemia, and treatment of CKD [29]. Retinal microvascular abnormalities are common, especially in patients with hypertension, renal vascular disease, and diabetes, and affect more than half of patients with renal failure [30]. Although our study did not focus on the exact cause of visual impairment, it can be assumed that in most patients visual impairment had a vascular basis. The strong relationship between vascular stiffness (PWV) and the risk of falling in the PPA test can be indirectly explained by the limitation of contrast discrimination.

It is worth mentioning mineral and bone disorders (CKD-MBD) common in patients with CKD. Many studies indicate a correlation between the degree of arterial calcification and the resulting arterial stiffening and the risk of cardiovascular complications, such as left ventricular dysfunction with concomitant heart failure and cardiac arrhythmias [31]. These factors also contribute to the increased risk of falls in these patients.

The relationship between eye diseases such as glaucoma and cataracts in CKD patients is unclear [32], but higher mean blood pressure and higher pulse pressure were associated with faster visual field progression in glaucoma patients [33].

Based on our observations, referring patients for preventive vision assessments and potential vision correction is advisable to help reduce the risk of falls. Additionally, this approach could provide valuable insights for modifying home environments, such as improving apartment lighting.

Our study has some limitations that should be acknowledged. First, it was conducted at a single center with a relatively small sample size, which may limit the generalizability of the findings. Second, although this was a prospective observational study, we did not reassess baseline parameters (e.g., S-PPA scores, hemodynamic measurements) during the follow-up period, which could have provided additional insights into the progression of motor coordination disorders and vascular dysfunction. Third, we did not perform detailed ophthalmologic evaluations to determine the specific causes of visual impairment (e.g., retinopathy, cataracts, or glaucoma), despite identifying contrast sensitivity as an independent predictor of falls.

Additionally, potential confounding factors—such as medication use (e.g., antihypertensives), cognitive function, or environmental hazards—were not systematically analyzed, though they may influence fall incidence. Finally, while arterial stiffness (PWV) showed a strong correlation with coordination impairments, the study design does not establish causality. Longitudinal or interventional studies are needed to confirm whether reducing arterial stiffness improves motor function in CKD patients.

## 5. Conclusions

In this prospective study, we demonstrated that impaired motor coordination and an increased risk of falls in CKD patients are strongly associated with cardiovascular dysfunction and vascular stiffening. Specifically, reduced arterial compliance—reflected by elevated pulse wave velocity (PWV) and augmentation index—was linked to greater postural instability, slower reaction times, and poorer muscle strength. We fund, that contrast sensitivity impairment emerged as an independent predictor of falls, underscoring the role of visual deficits in fall risk among this population. These findings highlight the need for multidisciplinary strategies in managing CKD patients, including:

– Regular assessment of motor coordination** (e.g., S-PPA) and vascular health (e.g., PWV) to identify high-risk individuals.
– Targeted interventions, such as vision correction, balance training, and optimization of cardiovascular therapy, to mitigate fall risk.
– Further research to explore whether reducing arterial stiffness (e.g., through pharmacologic or lifestyle interventions) improves physical function and reduces falls in CKD.

Future studies should incorporate comprehensive eye examinations to clarify the relationship between CKD-related visual deficits and fall risk.

## Figures and Tables

**Table 1:** Baseline clinical data and two-year follow-up data.

	Median of PPA Score < 3.36 No. 67	Median of PPA Score ≥ 3.36 No. 65	*p*
Demografic			
Age (y.) (±SD)	57.95 ± 14.2	71.66 ± 11.5	<0.01
Female (%)	21 (31)	33 (65)	0.16 *
Weight (kg) (±SD)	83 ± 19.49	75.18 ± 16.44	<0.05
Height (m) (±SD)	1.70 ± 0.09	1.65 ± 0.09	<0.01
BMI (/kg/m^2^) (±SD)	28.30 ± 5.87	27.2 ± 5.02	0.26 *
Cause of CKD			
HTN and DM (%)	27 (40)	30 (46)	>0.05 *
GN (%)	21 (31)	17 (26)	>0.05 *
others (%)	19 (28)	18 (28)	>0.05 *
Comorbidity			
CCI (point) (±SD)	4.48 ± 2.7	5.86 ± 2.27	<0.01
Number of HD patients with history of CV disease (%)	13 (19)	24 (37)	>0.05 *
Number of HD patients (%)	17 (25)	23 (35)	>0.05 *
Smoking (pack/y) (±SD)	7.36 ± 14.93	7.80 ± 13.68	>0.05 *
Mobil-O-Graph^®^ blood pressure monitor parameters			
pSBP (mmHg) (±SD)	135.5 ± 21.39	142.23 ± 28.12	>0.05
pMBP (mmHg) (±SD)	109.09 ± 15.95	109.66 ± 20.06	>0.05
pDBP (mmHg) (±SD)	87.07 ± 15.07	81.63 ± 15.66	>0.05
pPP (mmHg) (±SD)	48.09 ± 18.27	59.86 ± 20.41	<0.01
HR (beat/min) (±SD)	72.67 ± 13.69	73 ± 16.12	>0.05
AIx@75 (%) (±SD)	16.29 ± 15.34	22.51 ± 14.48	<0.05
Heart stroke volume (mL) (±SD)	74.66 ± 18.26	75.94 ± 17.23	>0.05
CO (mL/min) (±SD)	5.26 ± 0.99	5.38 ± 1.05	>0.05
Augmentation pressure (mmHg) (±SD)	6.85 ± 7.65	12.08 ± 11.52	<0.01
PWV (m/s) (±SD)	8.35 ± 1.95	10.65 ± 2.31	<0.01
QRISK3—risk of having a heart attack or stroke within the next 10 years (%)			
QRISK3 (%) (±SD)	22.54 ± 15.05	32.09 ± 15.71	<0.01
QRISK3—the relative risk (±SD)	5.01 ± 5.17	2.65 ± 3.33	<0.01
Physiological Profile Assessment			
Fall risk score (Z score) (±SD)	1.86 ± 0.98	5.41 ± 1.98	<0.01
Fall risk score (point) (±SD)	3.19 ± 0.98	4.53 ± 0.50	<0.01
Melbourne edge test (dB) (±SD)	17.10 ± 2.26	12.5 ± 3.88	<0.01
Proprioception (%) (±SD)	2.05 ± 6.01	3.33 ± 4.29	>0.05
Isometric knee extension test right (kg) (±SD)	24.50 ± 10.07	16.75 ± 7.43	<0.01
Isometric knee extension test left (kg) (±SD)	24.12 ± 10.44	15.47 ± 7.06	<0.01
The hand reaction time test (s) (±SD)	244.92 ± 46.90	402 ± 162	<0.01
The postural sway test (cm^2^) (±SD)	37.80 ± 86.01	200.35 ± 235.57	<0.01
30-second sit-to-stand test (No. of repetitions) (±SD)	14.85 ± 6.64	8.95 ± 5.65	<0.01
Biochemistry			
Hemoglobin (g/dL) (±SD)	10.77 ± 1.59	10.65 ± 2.03	>0.05
Total protein (g/dL) (±SD)	6.04 ± 0.91	6.2 ± 0.74	>0.05
Albuminy (g/dL) (±SD)	3.40 ± 0.59	3.42 ± 0.48	>0.05
TC (mg/dL) (±SD)	205.94 ± 68.71	181.74 ± 61.31	>0.05
TG (mg/dL) (±SD)	186.90 ± 85.73	134.70 ± 71.50	<0.01
CRP (mg/L) (±SD)	12.94 ± 16.87	9.12 ± 13.73	>0.05
Ca++ (mg/dL) (±SD)	8.65 ± 1.07	8.67 ± 0.73	>0.05
Pi (mg/dL) (±SD)	5.52 ± 1.52	5.12 ± 1.61	>0.05
PTH (pg/mL)	467 ± 474	342 ± 443	>0.05
AP (U/L) (±SD)	117 ± 154	116 ± 93	>0.05
sCr (mg/dL)	4.63 ± 2.58	4.22 ± 2.27	>0.05
GFR (mL/min.)	22.39 ± 25.50	18.33 ± 13.99	>0.05
urea (mg/dL)	108.41 ± 42.93	114.57 ± 39.86	>0.05
2-years follow-up			
Overall number of falls in 2 years	11	73	
Number of patients who fell within 2 years (%)	8 (12)	41 (63)	<0.01 *
Number of patients who had a cardiovascular incident within 2 years (%)	2 (3)	10 (15)	<0.05 *
Number of deaths in 2 years	0 (0)	14 (22)	<0.01 *

Abbreviation: BMI, body mass index; HTN, hypertensive nephropathy; DM, diabetes mellites; GN, glomerulonephritis; CCI, Charlson comorbidity index; SBP, systolic blood pressure; MBP, mean blood pressure; DBP, diastolic blood pressure blood pressure; PP, pulse pressure; HR, heart rate; PWV, pulse wave velocity; AIx@75, augmentation index normalized with 75/minute heart rate; QRISK3, 10-year risk of cardiovascular accident; TC, total cholesterol; TG, triglycerides; CRP, C-reactive protein; Ca++, calcium; Pi, phosphate; PTH, parathormone; AP, alkaline phosphatase; sCr, serum creatinine; GFR, glomerular filtration rate. * indicates chi-square test.

**Table 2:** Univariate logistic regression. Variables associated with patients who have fallen (at least one event of falling during the two years of follow-up).

	OR	95%CI		*p*
Demographic				
Age 50–59 years	0.372	0.127	1.084	>0.05
Age 60–69 years	0.862	0.430	1.730	>0.05
Age 70–79 years	2.601	1.293	5.230	<0.01
>80 years	4.953	1.597	15.363	<0.01
Age (y.)	1.083	1.043	1.125	<0.01
Female (Y1)	1.767	1.222	2.555	<0.01
BMI (kg/m^2^)	1.017	0.953	1.085	>0.05
Comorbidity				
CCI (point)	1.290	1.106	1.505	<0.01
No. of CV disease in interview	1.405	0.905	2.181	>0.05
CV disease Y = 1	1.280	0.881	1.860	>0.05
Mobil-O-Graph^®^ blood pressure monitor parameters				
PP (mmHg)	1.470	1.188	1.820	<0.01
PWV (m/s)	1.036	1.012	1.061	<0.01
AIx@75 (mmHg) (%)	1.007	0.979	1.036	>0.05
QRISK3—risk of having a heart attack or stroke within the next 10 years (%)				
QRISK3 (%)	1.041	1.016	1.066	<0.01
Physiological Profile Assessment				
Z score	1.705	1.363	2.132	<0.01
Fall risk score	3.161	1.815	5.504	<0.01
Melbourne edge test (dB)	0.732	0.646	0.829	<0.01
The strength of the right hand grip (kg)	0.905	0.860	0.952	<0.01
The strength of the left hand grip (kg)	0.912	0.869	0.957	<0.01
Average response time (cs)	1.890	2.703	1.321	<0.01
Postural sway (cm^2^)	1.004	1.006	1.001	<0.01
30-second sit-to-stand test (No.)	0.840	0.775	0.911	<0.01
Laboratory				
Hemoglobin (g/dL)	0.938	0.768	1.144	>0.05
Total protein (g/dL)	0.798	0.509	1.251	>0.05
Albuminy (g/dL)	0.709	0.354	1.421	>0.05
Pi (mg/dL)	0.779	0.582	1.044	>0.05
2-years follow-up				
Death during 2 years (Y1)	1.949	1.090	3.487	<0.01
CV incidents (Y1)	1.135	0.347	3.713	>0.05

Abbreviations: BMI, body mass index; CCI, Charlson comorbidity index; CV, cardiovascular; PP, pulse pressure; PWV, pulse wave velocity; QRISK3, 10-year risk of cardiovascular accident; Pi, inorganic phosphorus.

**Table 3:** Multivariate logistic regression. Variables correlated to patients who have fallen (incident of fall during the 2 years of follow-up).

	adj.OR	95%CI		*p*	Walda	Estimate
Age 50–59 years	1.034	0.162	6.578	>0.05	0.001	0.033
Age 60–69 years	3.253	0.867	12.211	>0.05	3.055	1.180
Age 70–79 years	0.779	0.206	2.948	>0.05	0.135	−0.249
>80 years	0.071	0.004	1.338	>0.05	3.116	−2.642
Female (Y1)	2.009	0.877	4.602	>0.05	2.718	0.697
Z score	1.085	0.610	1.931	>0.05	0.078	0.082
PWV (m/s)	1.347	0.809	2.243	>0.05	1.312	0.298
QRISK3 (%)	1.050	0.983	1.121	>0.05	2.126	0.049
Fall risk score	0.677	0.221	2.072	>0.05	0.467	−0.390
Melbourne edge test (dB)	0.712	0.535	0.948	<0.05	5.422	−0.340
The strength of the right hand grip (kg)	0.938	0.852	1.033	>0.05	1.675	−0.064
Average response time (cs)	1.001	0.995	1.008	>0.05	0.192	0.001
30-s sit-to-stand test (No.)	1.005	0.866	1.166	>0.05	0.004	0.005
CCI (point)	1.041	0.777	1.394	>0.05	0.073	0.040

Abbreviations: CCI, Charlson comorbidity index; PWV, pulse wave velocity; QRISK3, 10-year risk of cardiovascular accident.

## Data Availability

The data presented in this study are available from the corresponding author upon request.
